# Citizenship and recovery: two intertwined concepts for civic-recovery

**DOI:** 10.1186/s12888-015-0420-2

**Published:** 2015-03-04

**Authors:** Jean-François Pelletier, Marc Corbière, Tania Lecomte, Catherine Briand, Patrick Corrigan, Larry Davidson, Michael Rowe

**Affiliations:** 1 Department of Psychiatry, University of Montreal, Montreal, Canada; 2 Centre de recherche de l’Institut universitaire en santé mentale de Montréal (U.218), 7401, Hochelaga Street, Montreal, QC H1N 3M5 Canada; 3 Department of Psychiatry, Yale School of Medicine, New Haven, USA; 4 School of rehabilitation, University of Sherbrooke, Sherbrooke, Canada; 5 Department of Psychology, University of Montreal, Montreal, Canada; 6 School of rehabilitation, University of Montreal, Montreal, Canada; 7 Lewis College of Human Sciences, Illinois Institute of Technology, Chicago, USA; 8 Department of Education, Université du Québec à Montréal, Montreal, Canada

**Keywords:** Citizenship, Recovery, Civic-recovery, Tool validation, Recovery Assessment Scale, Citizenship Measure

## Abstract

**Background:**

Validation of the psychometric properties of a new measure of citizenship was required for a research project in the province of Quebec, Canada. This study was meant to study the interplay between recovery- and citizenship-oriented supportive employment. As recovery and citizenship were expected to be two related concepts, convergent validity between the Citizenship Measure (CM) and the Recovery Assessment Scale (RAS) was tested.

**Methods:**

Study objectives were to: 1) conduct exploratory factor analyses on the CM and confirmatory factor analysis on the RAS tools (construct validity), 2) calculate Cronbach’s alphas for each dimension emerging from objective 1 (reliability), and 3) calculate correlations between all dimensions from both tools (convergent validity). Data were collected from 174 individuals with serious mental illness, working in social firms. Serious mental illnesses include major depression, schizophrenia, bipolar disorder, obsessive compulsive disorder, panic disorder, post traumatic stress disorder and borderline personality disorder.

**Results:**

Five factors emerged from the exploratory factor analysis of the CM, with good reliability. Confirmatory factor analyses showed that the short and the long versions of the RAS present satisfactory results. Finally, the correlation matrix indicated that all dimensions from both tools are significantly correlated, thus confirming their convergent validity.

**Conclusions:**

This study confirms the validity and reliability of two tools, CM and RAS. These tools can be used in combination to assess citizenship and recovery, both of which may be combined in the new concept of civic-recovery.

## Background

The recovery approach has gained traction in mental health policy throughout the English-speaking world, and much effort is going into the transformation of services and systems to achieve recovery-oriented outcomes [1,2]. Beyond reduction or remission of psychiatric symptoms, recovery-oriented mental health policies and systems not only help the individuals with mental illness to live in their community – to be *in* the community – but also aim at enabling them to become and remain members *of* the community [3].

Several evidence-based interventions exist that focus on the psychiatric rehabilitation and social inclusion of people living with mental illness. Social interaction, which is essential to community membership, involves the development and maintenance of reciprocal relationships between members of a community in which each of its components are co-citizens to each other. Citizenship relates to the strength of people’s connections to the rights, responsibilities, roles, and resources that society offers to people through public and social institutions, and relationships involving close ties, supportive social networks, and associational life in one’s community [4,5].

As early as 1994, in the early ages of the recovery movement [6], Fisher developed an empowerment model of recovery based on the principles that emerged from the experiences of consumers in recovery. Among those principles is *Personhood*: “We are full human beings and deserve respect and full citizenship” ([7], p. 914). More recently, Davidson et al. suggest that, as a sense of empowerment and control over one’s life emerges, people in recovery may start to demand the same rights and responsibilities as other citizens [8,9]. Supporting people with mental illness in exercising the rights and responsibilities of citizenship might be a pre-condition for their recovery, not an eventual reward contingent on the person overcoming his or her disability first. Indeed, almost from inception, recovery proponents have alluded to citizenship, albeit rarely defining the term and without attempts to measure it empirically. One of the aims of this paper is to contribute to the filling of that gap by comparing two measurement tools: one designed to assess citizenship, the other one to assess recovery from the perspective of persons in recovery.

Generally speaking, two portrayals of recovery stand out amidst the diversity of views: *restoration of functioning* and *deepening wellness* [10]. When recovery is mainly seen as symptom management, the primary focus of personal choice and responsibility in the process of recovery becomes seeking and complying with treatment. Such a “clinical” model does include social functions, but from a professional point of view. Instead of focusing primarily on symptom relief and management, a second view casts a wider spotlight on restoration of self-esteem and identity and on attaining meaningful roles in society [11]. While the clinical-recovery model has focused upon the remission of symptoms and restoration of functioning, a rehabilitative view of recovery has been a more subjective and consumer-oriented concept that focuses on the full lives that are lived in the face of, or despite, enduring disability. This second axiom of recovery derives from the Mental Health Consumer/Survivor Movement, and refers to a person’s rights to self-determination and inclusion in community life regardless of disability status.

For a research project meant to study the interplay between recovery- and citizenship-oriented supportive employments, validation of the psychometric properties of a recently developed measure of citizenship was required. As citizenship and recovery were expected to be related concepts, convergent validity between the Citizenship Measure (CM) and the Recovery Assessment Scale (RAS) was tested.

Although the recovery literature has now and then alluded to citizenship, clinicians’ and scientists’ references or pledges to citizenship have been sparse and, generally, vague. Several tools have been developed to assess recovery [12], but until very recently no measure was available to assess citizenship from the perspective of persons in recovery. In a review of the literature [13] based on a MEDLINE search with the key words “citizenship”, “measure”, and “mental health”, the CM is the only referenced tool specifically designed to assess the degrees to which individuals with psychiatric disorders perceive themselves to have full citizenship [14]. On the other hand, the search with “recovery”, “measure”, and “mental health” yielded 392 references. Among those is the RAS, which was developed as an outcome measure for program evaluation [15]. In this paper we explore the RAS and the CM, which was developed, too, as an outcome measure.

Salzer and Brusilovskiy have recently published an in-depth review of the quantitative properties of the RAS, based on 77 articles that included psychometric data. They found that these studies indicate very good results for internal consistency, test-retest reliability, and internal reliability [16]. It has however not yet been validated in French. Among the tools that were developed to empirically assess recovery, the RAS has been the most published [17], whereas the CM is the only published tool developed to assess citizenship. Although citizenship is a distinct concept, it overlaps somewhat with the Mental Health Consumer/Survivor Movement’s view of recovery. As such, a citizenship measure such as CM should demonstrate convergent validity with a recovery measure like the RAS.

Therefore, this study consists of the validation of the French CM and RAS. The objectives are threefold: 1) to conduct factor analyses on both tools (i.e., construct validity), more precisely: 1.1) exploratory factor analysis on the CM and 1.2) confirmatory factor analysis on the RAS, 2) to calculate Cronbach’s alphas for each dimension emerging from the objective 1 (i.e., reliability), and 3) to calculate correlations between all dimensions from both tools (i.e., convergent validity). Convergent validity is a series of tests to see whether constructs that are expected to be related are, in fact, related [18]. Thus we chose to look at the CM and the RAS together. The alphas for the factors of the original RAS ranged from 0.74 to 0.87 [15], and the alphas for the English version of the CM ranged from 0.56 to 0.86 [O’Connell et al, Reliability and validity of a newly developed measure of citizenship among persons with mental illnesses, submitted].

## Methods

Two translators translated the CM and RAS from English to French separately and then two other translators translated each of the measurements backward from French to English for discussion with the English speaking authors of the CM and RAS. For this study we thus used translation-back-translation procedures [19] to translate into French both the CM (M.R., co-author) and the RAS (P.C., co-author) and to explore possible convergent validity between the measures of citizenship and recovery. The primary aim of this paper is to evaluate the psychometrics of the new French-versions of the CM and RAS among French speaking research participants in the province of Quebec, Canada.

The CM is a scale of 46 items that are rated on a 5-point Likert scale (5 = strongly agree; 1 = strongly disagree). Multidimensional scaling and hierarchical cluster analysis revealed seven primary domains of citizenship: *personal responsibilities*; *government and infrastructure*; *caring for self and others*; *civil rights*; *legal rights*; *choices*; and *world stewardship* [14].

The RAS has been used to assess various aspects of recovery from the perspective of persons with serious mental illness and with a particular emphasis on hope and self-determination. The original instrument comprises 41 items, and a shorter version containing 24 items is also available [20]. We used both versions, for which all items are also rated on a 5-point Lickert scale (5 = strongly agree; 1 = strongly disagree). The RAS covers five domains: *personal confidence*; *willingness to ask for help*; *goal and success orientation*; *reliance on others*; and *no domination by symptoms*.

A total of 183 individuals provided usable data; missing values for some items have lowered the *n* to 174. These participants, all people with serious mental illness, were involved in governmental work integration programs and participated in the abovementioned larger study, with a majority of them receiving the equivalent in Quebec of the Social Security Disability Insurance in the USA (SSDI). Serious mental illnesses include major depression, schizophrenia, bipolar disorder, obsessive compulsive disorder, panic disorder, post traumatic stress disorder and borderline personality disorder. This study was approved by the Institutional Review Board of *Institut universitaire en santé mentale de Montréal* (affiliated with the University of Montreal) and written informed consent for participation in the study was obtained from participants. Fifty-four percent were males (*n* = 94), and the mean age was 45.5 (SD = 10). Approximately one half (*n* = 83) self-reported a diagnosis of schizophrenia spectrum disorders. Anxiety disorder (*n* = 22), bipolar disorder (*n* = 23) and major depression (*n* = 22) were each mentioned by about 13% of participants. Another 4% mentioned having a personality disorder (*n* = 8). The diagnosis was unknown or unspecified among 9% of participants (*n* = 16). All participants were French speaking.

### Data analysis

As suggested by Costello and Osborne [21], a principal axis factoring method of extraction (PAF) with oblique (oblimin) rotation was applied to the CM. Different indices were considered such as scree plot, eigenvalues, percentage of total variance, and item loadings on each emerging dimension. Confirmatory factor analyses (CFA) were carried out on the French translation of RAS – long and short versions – which consists of items spread over the following five dimension scales: (1) *personal confidence*; (2) *willingness to ask for help*; (3) *goal and success orientation*; (4) *reliance on others*; and (5) *no domination by symptoms*.

CFA procedure permits the evaluation of the model’s fit with the empirical data by taking into account various statistical indices. Using the EQS software [22], the estimation method maximum likelihood-robust was carried out to evaluate the model with five subscales, considering the 24-item short version or the long version (40 out of 41 items). Note that, for the long version, we did not include the item that was not part of any subscale (*I am a better person than before my experience with mental illness*). The Satorra-Bentler scaled chi-square index method was used to control for violation of the assumption of multivariate normality, since this index integrates a scale that corrects chi-square statistics.

The internal consistency of each of the CM and RAS scales, from the exploratory factor analysis (CM) and confirmatory factor analysis (RAS) results respectively, was assessed using Cronbach’s alpha [23]. Pearson correlations were calculated between the dimensions of the CM and the RAS to assess the tools’ convergent validity. Beaver et al. consider that when the factors load moderately (.40 or higher), as is the case here (see below), a sample size of 150 or more is needed to be confident in the results at such an initial stage [24].

## Results

A PAF was carried out on the CM (46 items). The results indicated a value of 35.9% of the total variance (the total variance would have been 49.2% with principal components analysis combined to orthogonal varimax rotation). After oblimin rotation plus multiple test runs for information on how many meaningful factors might be in this data set, the dimensions showed a very well distributed variance on the five dimensions (Table 1). This type of rotation was chosen because oblique rotation will reproduce an orthogonal solution but not vice versa [21]. Consequently, the PAF-oblimin combination revealed 23 items spread out on the following five dimensions: *Self-determination* (6 items, Cronbach’s alpha = 0.67), *respect by others* (4 items, Cronbach’s alpha = 0.74), *involvement in community* (4 items, Cronbach’s alphas = 0.65), *basic needs* (5 items, Cronbach’s alpha = 0.60), and *access to services* (4 items, Cronbach’s alpha = 0.60) (Table 1).

**Table 1:** **Factor structure of the Citizenship Measure (N = 174)**

	Factors				
Dimensions and items of the citizenship scale	1	2	3	4	5
**Basic needs** (n = 5 items; **α** = .60)					
**-** Your basic needs are met	.55				
**-** You do things to take care of your home	.42				
**-** You are safe in your community	.39				
**-** There are laws that will protect you	.39				
**-** You have or would have access to employment	.31				
**Involvement in community** (n = 4 items; **α** = .65)					
**-** You are include in your community		.60			
**-** You have responsibilities to others in the community		.58			
**-** You can influence your community or local government		.54			
**-** You have knowledge about your community		.49			
**Self-determination** (n = 6 items; **α** = .67)					
**-** You or your family have choices in education			.58		
**-** You stand up for what you believe in			.56		-.31
**-** You have the right to be in a relationship with a partner of your choice			.43		
**-** You have privacy			.40		
**-** You have the right to be disagree with others			.32		
**-** You can make choices about how you spend your money			.30		
**Basic needs** (n = 5 items; **α** = .60)					
**-** You have access to adequate healthcare				-.67	
**-** You have or could have access to adequate and affordable housing				-.54	
**-** You would have access to public assistance, if needed	-.32		.30	-.49	
**-** You have choices in your mental healthcare				-.32	
**Respect by others** (n = 4 items; **α** = .74)					
**-** You are treated with dignity and respect					-.69
**-** You feel accepted by you					-.63
**-** You listen to you					-.52
**-** You personal decisions and choices are respected					-.43
Eigenvalues	4.9	1.9	1.7	1.5	1.4
Variance after rotation	18.6	5.6	4.8	3.6	3.3

Two confirmatory factor analyses were carried out on the following five RAS subscales (short and long versions): *Personal confidence and hope* (9 and 12 items), *willingness to ask for help* (3 and 5 items), *goal and success orientation* (5 and 8 items), *reliance on others* (4 and 5 items), and *no domination by symptoms* (3 and 10 items). Both confirmatory factor analyses did yield a satisfactory model adjustment (Table 2). In fact, the four fit indices, Non-Normed Fit Index (NNFI), Comparative Fit Index (CFI), robust CFI, and Incremental Fit Index (IFI), were above the threshold of .90. The respective values for root mean square error of approximation (RMSEA) (.03) and chi-square/df (1.13 or 1.14) are also satisfactory for both versions of the RAS [25,26]. Results indicated an acceptable factor solution, with the Cronbach’s alphas for these five factors being all adequate and ranging from 0.74 to 0.87. Although the two versions are satisfactory, a parsimonious model is preferable to one that is more complex. Among cross validation indices [27], the Akaike Information Criterion (AIC) [28] can be used with the EQS software package to show which model is the most parsimonious. No specific threshold is recommended for this AIC index. However, lower values indicate better fit with the data and thus the model that has the lowest AIC coefficient is the model that should be recommended. In this case, the AIC coefficient for the short 24-item French RAS is 6.19, whereas the AIC coefficient for the long 40-item French RAS is 50.19, as shown in Table 2.

**Table 2:** **Confirmatory factor analyses results of the Recovery Assessment Scale (N = 174)**

Models	Adjustment fit indices							
	df	χ ^ 2 ^	χ ^ 2 ^ /df	NNFI	CFI rubost	IFI	RMSEA	AIC
**Recovery**								
*Five dimensions*								
**M1-Short version with all factors corrected**	242	273.36	1.13	.95	.96	.96	.03 (confidence interval = .01-.04)	6.19
(24 items)								
**M2 - Long version with all factors associated**	730	832.05	1.14	.91	.92	.92	.03 (confidences interval = .02 - .04)	50.19
(40 items)								

Note: NNFI = Non NormedFit Index; CFI = Comparative Fit Index; IFI = Bollen Incremental Fit Index; RMSEA = Root Mean Square Error of Approximation; AIC = Akaike Information Criterion. The model in bold (M1) corresponds to the best adjusted one.

The internal consistency coefficients for each scale pertaining to CM, and RAS questionnaires are all satisfactory (given the small number of items for some scales) and vary from .60 to .80 (Table 3). For the five dimensions of the CM and RAS respectively, the correlations were from .41 to .77 (*p* < .01) and from .18 to .46 (*p* < .01). Finally, the correlation coefficients between all dimensions (RAS and CM), were over .21 (*p* < .01), and ranging from .21 to .52 (i.e. RAS – *goal and success orientation* associated with CM – *basic needs*).

**Table 3:** **Correlations among dimensions – citizenship and recovery measures**

	*1*	*2*	*3*	*4*	*5*	*6*	*7*	*8*	*9*	*10*
1. CM - Self-determination	*.67*									
2. CM - Respect by others	.37	*.74*								
3. CM - Involvement in community	.18	.30	*.65*							
4. CM - Fundamental needs	.36	.46	.32	*.60*						
5. CM - Access to services	.38	.29	.26	.28	*.60*					
6. RAS - Personal confidences	.33	.46	.36	.48	.27	*.86*				
7. RAS - Willingness to ask for help	.34	.45	.33	.43	.38	.75	*.61*			
8. RAS - Goal and success orientation	.48	.44	.30	.52	.33	.77	.67	*.80*		
9. RAS - Reliance on others	.27	.40	.39	.30	.22	.51	.46	.49	*.60*	
10. RAS - No domination by symptoms	.37	.21	.37	.23	.37	.55	.47	.58	.41	*.77*

Note: N = 174. Cronbach’s alpha in italic along the diagonal. All correction coefficients are p < 0.01.

RAS = Recovery Assessment Scale.

CM = Citizenship measurement.

## Discussion

With respect to the value of the alpha, Streiner and Norman [29] mention that the alpha should be between 0.70 and 0.90 to have a good internal consistency for the evaluated conceptual dimension. However, if the number of items is inferior to 5, it is possible to obtain a low alpha coefficient (around 0.65) whereas if the number of items is superior to 10, the value of the alpha is higher (around 0.90). Consequently, according to the number of items included in a conceptual dimension, alpha coefficients can vary considerably [30], though DeVellis [31] mention that an alpha inferior to .60 is not acceptable regardless of the number of items. If we consider the number of items included in conceptual dimensions or subscales from the RAS and CM, all alpha values in our study are satisfactory or acceptable.

Therefore, the construct validity and the reliability for each tool, the French CM and RAS, are satisfactory. This study demonstrates the empirical relationship between citizenship and recovery, supporting the convergent validity of the CM measure. In effect, our findings suggest a correlational relationship between the five dimensions of the CM and RAS respectively. Citizenship and recovery would appear to be two intertwined and complementary concepts. This does not suggest, however, a causal relationship between the two concepts. We cannot predict, for instance, that a recovery-oriented program would necessarily imply good outcomes in terms of citizenship for people with mental illness, nor vice-versa.

In terms of implications for practice, what we do argue is that, for future research, more precision is necessary as the idea of recovery remains controversial and as there remains little consensus on what recovery means, especially when an individual’s understanding of his/her own recovery changes over time [32,33]. Therefore, the global aim of this paper is to contribute to the field by proposing a new angle of analysis, in complementarity with the clinical and rehabilitative views.

To prevent what Slade et al. have called an *abuse* of recovery [34], more clarity in the field is warranted, with authors specifying for instance which view of recovery they are referring to. In effect, researchers and scholars who write about recovery are rarely explicit about whether or not they are writing from the clinical-recovery or the rehabilitative-recovery views [35]. The latter has focused more on the subjective sense of living a life in recovery and on changing mental health care than on ensuring that people in recovery are and remain full members *of* the community – not just *in* the community. Citizenship, with community membership at its core, can help to tease apart these various aspects of recovery. As recovery presupposes interaction factors relating to the person and the environment, plus elements from professional interventions [9], it might be appropriate to combine the short CM and the short RAS (given that the short RAS is the best adjusted model) as an outcome measure for recovery- and citizenship-oriented services. There would be 10 dimensions to such a composite index – in alphabetical order: *Access to services; Basic needs; Goal and success orientation; Involvement in community; No domination by symptoms; Personal confidence and hope*; *Reliance on others*; *Respect by others; Self-determination;* and *Willingness to ask for help.* Each item can be uniformly answered with a 5-point Likert scale, for a total of 47 questions.

Undoubtedly, there is an increasing global commitment to recovery as *the* expectation for people with mental illness. Recovery is a process in which the person engages to figure out how to manage and live with his or her disorder. Recovery and citizenship are neither things that providers can do to, or for people with mental illness, nor things that can be promoted after or separate from treatment and other clinical services. Using the CM-RSA combination as an outcome measure for the existing services may be one place to start, while pursuing new evidence from further research on citizenship and community membership for people with mental illness.

### Limitations

Among the limits inherent to this study is the fact that participants were already involved in a process of reintegration into work and therefore could be already in the process of recovery towards full citizenship. But this might also be an advantage because such persons may feel that they do have an opinion to share about the content of items. Another limitation is that the sample consists only of French speaking participants and our civic recovery combination pertains to findings for the French CM and RAS measures. We suggest to test the civic-recovery composite index factor solutions with other people. Also, as our hypothesis is that recovery and citizenship are two related constructs, convergent validity between the CM and RAS was explored. Citizenship and recovery may be related, but they certainly are not synonymous with one another, just like recovery vs self-esteem [36]. More research is needed to assess if, and to what extent these would be different constructs (concurrent validity). Also of importance is to underscore the preliminary nature of these findings, given the small sample size.

## Conclusion

This study provided further validation for the Citizenship Measure (CM) and the Recovery Assessment Scale (RAS). Exploratory Factor Analysis and Confirmatory Factor Analyses’ results supported the construct validity and reliability for each dimension or subscale. Significant correlations were also found between these scales and their subscales, thus confirming the convergent validity of both tools. This convergence suggests that the CM and RAS can be used in complementarity of current axioms of recovery to assess civic-recovery as an outcome measure for program evaluations, but more research is needed to explore this new construct.
